# The effect of multimorbidity patterns on physical and cognitive function in diabetes patients: a longitudinal cohort of middle-aged and older adults in China

**DOI:** 10.3389/fnagi.2024.1388656

**Published:** 2024-05-14

**Authors:** Xieting Zhou, Juan-Juan Qin, Hang Li, Jiyu Chen, Qing Zhang, Xujun Ye

**Affiliations:** School of Nursing, Department of Geriatric, Zhongnan Hospital of Wuhan University, Wuhan, China

**Keywords:** diabetes, multimorbidity, cognitive function, disability, physical function

## Abstract

**Background:**

The prevalence of diabetes has increased rapidly, and comorbid chronic conditions are common among diabetes patients. However, little is known about the pattern of multimorbidity in diabetes patients and the effect on physical and cognitive function. This study aimed to assess the disease clusters and patterns of multimorbidity in diabetes patients using a novel latent class analysis (LCA) approach in middle-aged and older adults and explore the association between different clusters of multimorbidity in diabetes and the effect on physical and cognitive function.

**Methods:**

This national observational study included 1,985 diabetes patients from the four waves of the China Health and Retirement Longitudinal Study (CHARLS) in 2011 to 2018. Thirteen chronic diseases were used in latent class analysis to identify the patterns of multimorbidity in diabetes, which span the cardiovascular, physical, psychological, and metabolic systems. Cognitive function is assessed via a structured questionnaire in three domains: memory, executive function, and orientation. We combined activities of daily living (ADL) with instrumental activities of daily living (IADL) to measure physical function. Linear mixed models and negative binomial regression models were used to analyze the association between patterns of multimorbidity in diabetes and the effect on cognitive function and disability, respectively.

**Results:**

A sample of 1,985 diabetic patients was identified, of which 1,889 (95.2%) had multimorbidity; their average age was 60.6 years (standard deviation (SD) = 9.5), and 53.1% were women. Three clusters were identified: “cardio-metabolic” (*n* = 972, 51.5%), “mental-dyslipidemia-arthritis” (*n* = 584, 30.9%), and “multisystem morbidity” (*n* = 333, 17.6%). Compared with diabetes alone, the “multisystem morbidity” class had an increased association with global cognitive decline. All patterns of multimorbidity were associated with an increased risk of memory decline and disability; however, the “multisystem morbidity” group also had the strongest association and presented a higher ADL-IADL disability (ratio = 4.22, 95% CI = 2.52, 7.08) and decline in memory Z scores (*β* = −0.322, 95% CI = −0.550, −0.095, *p* = 0.0058).

**Conclusion:**

Significant longitudinal associations between different patterns of multimorbidity in diabetes patients and memory decline and disability were observed in this study. Future studies are needed to understand the underlying mechanisms and common risk factors for multimorbidity in diabetes patients and to propose treatments that are more effective.

## Introduction

Diabetes is one of the major challenges for healthcare systems worldwide. In 2021, approximately 537 million adults were found to live with diabetes worldwide and 6.7 million people died from diabetes (International Diabetes Federation, 2021). According to previous research, approximately 97.5% of adults with diabetes have at least one chronic condition, and as many as 88.5% have two or more concurrent chronic conditions. Multimorbidity is defined as the co-occurrence of at least two chronic conditions in the same individual (Xu et al., 2017; Skou et al., 2022), including hypertension and coronary heart disease, as well as diseases affecting the mental system, the nervous system, chronic kidney disease, and chronic lung disease. Having a multimorbidity further increases the complexity of treatment for diabetes patients and is associated with reduced quality of life, impaired functional status, and increased burden on limited healthcare resources (Glynn et al., 2011; Marcel, 2013; Fu et al., 2022).

Compared with non-diabetic patients, diabetes patients are more likely to develop multiple conditions (Piette and Kerr, 2006). This greater risk reflects the fundamental impact of extended exposure to elevated glucose and insulin resistance on multiple organ systems. Evidence suggests that multimorbidity is likely to impair physical and mental health outcomes (Chen et al., 2011; Koyanagi et al., 2018; Makovski et al., 2019; Pati et al., 2020). A US cross-sectional study showed that patients with comorbid depression and diabetes are at an increased risk for activities of daily living (ADL) disability compared to those with either depression or diabetes alone (Egede, 2004). A 40-month cohort study found that depression was associated with accelerated cognitive decline in diabetes patients in comparison to non-depressed patients with diabetes (Sullivan et al., 2013). However, these studies have focused on single specific comorbidities (such as depression), rather than patterns of comorbidities. A prospective study found that varying clusters of comorbidities led to different results than some specific disease groups (Aarts et al., 2011). Previous studies examining patterns of multimorbidity in diabetes patients have focused on a count of numbers of conditions related to diabetes and independent conditions (Halanych et al., 2007; Kerr et al., 2007; An et al., 2019). These findings suggested that the single-disease orientation of diabetes management programs and guidelines is unlikely to address the healthcare needs of patients with diabetes. A systematic review on the effectiveness of interventions for the management of multimorbidity concluded that interventions targeted at specific risk factors or at specific problems, such as with functional ability or the management of medicines, are more likely to be effective (Smith et al., 2012). Therefore, it is important to recognize patterns of multimorbidity in diabetes, along with how they associate with health outcomes.

Using data of the China Health and Retirement Longitudinal Study (CHARLS), including middle-aged participants and older adults, we explored the disease clusters and patterns of multimorbidity in diabetes patients. In particular, the present study aimed (a) to test whether multimorbidity in diabetes patients increases the risk of disability and (b) to determine whether specific cognitive domains are differentially affected by multimorbidity in diabetes patients.

## Methods

### Data

The China Health and Retirement Longitudinal Study (CHARLS) is an ongoing nationally representative survey that investigates the social, economic, and health statuses of middle-aged and older people aged 45 years and above in China (Zhao et al., 2014). The baseline survey was conducted in 2011 with 17,708 participants and is followed-up every 2 years. Four follow-up visits are available: 2011 (wave 1), 2013 (wave 2), 2015 (wave 3), and 2018 (wave 4). The CHARLS datasets can be downloaded at the CHARLS home page at http://charls.pku.edu.cn/en. The CHARLS survey project was approved by the Biomedical Ethics Committee of Peking University, and all participants were required to sign informed consent.

### Population

**Figure 1:**
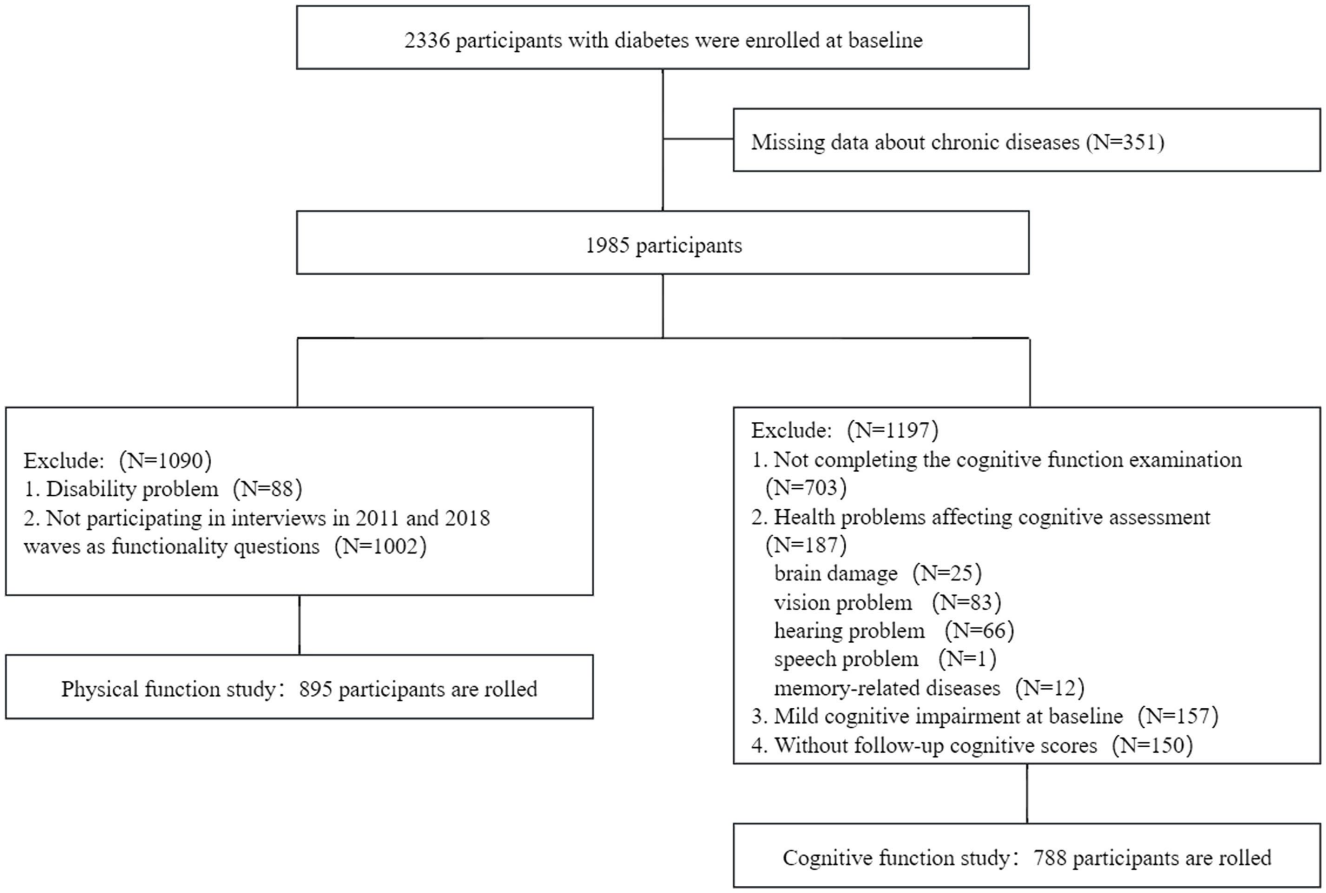
Flowchart of participant selection for the present study.

Of the 17,707 participants surveyed in 2011 and 2012, 2,336 (13.2%) participants with diabetes were included. A total of 351 participants with missing data about chronic diseases were excluded. For the cognitive function study, we excluded 1,197 participants based on the following criteria: (1) failure to complete the cognitive function examination at baseline; (2) presence of health problems affecting cognitive function, including brain damage, vision problem, hearing problem, speech problem, and memory-related diseases; (3) presence of mild cognitive impairment (MCI) at baseline; and (4) absence of follow-up cognitive function scores. The final sample included 788 participants with baseline data and at least one reassessment of cognitive function (wave 1 to wave 4). For the physical function study, participants with a disability at baseline or those who could not complete functionality questionnaires at the 2011 and 2018 waves were excluded. The final sample included 895 participants. The detailed flow chart of participant selection is shown in Figure 1.

### Chronic diseases and multimorbidity

Thirteen chronic diseases were modeled in this study, namely, hypertension, dyslipidemia, cancer, chronic lung disease, liver disease, heart disease, stroke, kidney disease, stomach or other digestive disease, psychiatric problems, arthritis, asthma, and depressive symptoms. Each participant’s disease status (yes or no) for a total of 13 non-communicable chronic diseases was confirmed by the patient’s self-report of a physician’s diagnosis “Have you been diagnosed with [conditions listed below, read one by one] by a doctor?” or in combination with medication data “Are you now taking any of the following treatments to treat […] or its complications (Check all that apply)? Taking Chinese traditional medicine, taking Western modern medicine, or other treatments?” in the 2011 CHARLS survey.

Diabetes is defined as fasting plasma glucose ≥126 mg/dL or HbA1c ≥ 6.5%, or current use of any treatment to control blood sugar, or any self-reported history of physician-diagnosed diabetes (American Diabetes Association Professional Practice Committee, 2021). Hypertension is defined as mean systolic blood pressure of ≥140 mmHg or mean diastolic blood pressure of ≥90 mmHg, or current use of antihypertensive drugs, or any self-reported history of physician-diagnosed hypertension (Chobanian et al., 2003). Dyslipidemia is defined as TC ≥ 240 mg/dL, or TG ≥ 200 mg/dL, or LDL-C ≥ 160 mg/dL, or HDL-C < 40 mg/dL, or taking any treatment to lower blood lipid levels, or having any self-reported history of physician-diagnosed dyslipidemia (Zhu et al., 2018). Multimorbidity is defined as the co-occurrence of at least two chronic conditions in the same individual.

### Depressive symptoms

The Center for Epidemiologic Studies Depressive Scale (CESD-10), a 10-item questionnaire, was used to measure depressive symptoms, which was highly validated for use in the general population (Chen and Mui, 2014). The respondents were asked to rate “how often you felt this way during the past week,” including their depressive behaviors and feelings such as depressive, loneliness, or fear. A four-scale metric was used to rate the CESD-10 answers, with the total score ranging from 0 to 30 points. Previous research studies have confirmed that a cutoff point of 10 is valid in identifying clinically depressive symptoms (Boey, 1999).

### Cognitive function

Cognitive function assessments, consisting of three domains, namely, memory, executive function, and orientation, were conducted in waves 1 to 4 in the CHARLS, using questionnaires that were adapted from the Telephone Interview for Cognitive Status (Fong et al., 2009; Ma et al., 2021). Memory was evaluated by immediate and delayed recall of 10 unrelated words. One point was given for each word recalled either immediately or delayed (0 to 10 points). The score of memory ranged from 0 to 20 points. Orientation was assessed by asking four questions based on the year, the month, the date of the month, and the day of the week (0 to 4 points). Executive function was evaluated by the Serial Sevens Test (0 to 5 points) and drawing the picture of two overlapping pentagons (0 or 3 points). The higher the cognitive scores, the better the cognitive function.

To evaluate the global cognitive function, Z scores were generated in each cohort, which has been widely accepted (Dregan et al., 2013; Zheng et al., 2018; Xie et al., 2019). First, the domain Z scores were generated by standardizing to the baseline. Each domain test score was subtracted by the mean and then divided by the standard deviation (SD) of the baseline domain scores. Second, the global Z scores of an individual at each wave were calculated from the mean score of the three domains by re-standardizing to the baseline.

Mild cognitive impairment (MCI) was defined according to aging-associated cognitive decline (AACD), namely, at least 1 standard deviation (SD) below the age standard in three domains, namely, memory, executive function, and orientation (Richards et al., 1999; Hu et al., 2022).

### Physical function

Participants were asked all ADL/IADL questions and were defined as having a specific ADL/IADL disability if they answered as follows: (1) I have difficulty but can still do it, (2) I have difficulty and need help, or (3) I cannot do it. The ADL index, which includes dressing, bathing, eating, going to bed, and using the toilet or controlling incontinence, represents the count of ADL disabilities for each participant (range 0–6; a higher number indicates higher ADL disability) (Katz, 1963). The IADL index, which includes doing housework, preparing meals, shopping, managing money, or taking medications, represents the count of IADL disabilities for each participant (range 0–5; a higher number indicates higher IADL disability) (Lawton and Brody, 1969). The ADL–IADL index was generated by summing the ADL and IADL index scores (range 0–11), which might capture a greater range of functional disability prevalence and has been previously validated (Spector and Fleishman, 1998; Thomas et al., 1998; Liang et al., 2008).

### Covariates

Sociodemographic characteristics and health-related factors, which were shown by previous studies to be associated with diabetes and cognitive function, were selected for our analyses. Sociodemographic characteristics included age (years), gender (male or female), education (illiterate, primary school, middle school, high school/vocational high school, and junior college or above), and marital status (married, cohabitating, separated/divorced/ widowed, and never married). Health-related factors included ever smoking (yes or no), ever drinking (yes or no), and body mass index (BMI), which is defined as the weight (kg) divided by the square of height (m). Blood data were also selected, including blood urea nitrogen (BUN), glucose, creatinine, glycated hemoglobin, total cholesterol, HDL-cholesterol, LDL-cholesterol, triacylglycerol, C-reactive protein (CRP), uric acid, and cystatin C.

### Statistical analysis

The results are presented as the mean ± SD or the median with the interquartile range (IQR) for continuous variables and numbers (percentage) for categorical variables.

Latent class analysis (LCA) was conducted to identify patterns of multimorbidity in the 1,889 diabetes participants who were defined with a multimorbidity at baseline. Thirteen chronic diseases (hypertension, dyslipidemia, cancer, chronic lung disease, liver disease, heart disease, stroke, kidney disease, stomach or other digestive disease, psychiatric problems, arthritis, asthma, and depressive symptoms) were used as observed indicators. We first tested increasingly complex models, beginning with two classes and ending with five classes. The best-fit model was determined using the Bayesian information criterion (BIC), the Akaike information criterion (AIC), and the Entropy Index (Seol and Chun, 2022). Lower values of BIC and AIC indicate better fit, whereas Entropy Index (0 to 1) represented the precision of the classification degree. The closer the value is to 1, the more accurate the classification is (Lubke and Muthén, 2007). The Lo–Mendell–Rubin likelihood ratio (LMR LR) test confirmed that the number of classified layers was the optimal value. We finally presented clusters ordered by descending prevalence and named each latent class (“cardio-metabolic” class, “mental-dyslipidemia-arthritis” class, and “multisystem morbidity” class) according to the most prevalent diseases (Bayes-Marin et al., 2020).

Missing data in the covariates were handled using multiple imputation by chained equations. The imputation model included all the variables used in the regression models. All analyses were conducted with R, version 4.2.2. LCAs were performed using Mplus.

Linear mixed models were used to evaluate longitudinal associations between different patterns of multimorbidity in diabetes patients and decline in cognitive Z scores. In the two models that we constructed, the intercept was fitted as random effects to account for interindividual differences at the baseline and the change in cognitive function over the follow-up period. The first model included the diabetes–multimorbidity group as the fixed-effect component. The second model was adjusted for age, gender, time (wave 1 to wave 4), education, marital status, ever smoking, ever drinking, BMI, and biomarkers.

Given the overdispersion of the outcome variable, we used negative binomial regression models to investigate the incremental burden of disability associated with patterns of multimorbidity in diabetes patients. The following models were tested: (1) unadjusted, (2) minimally adjusted (age, gender, education, and BMI), and (3) fully adjusted. The dependent variable was the ADL–IADL index. We compared the ADL–IADL indices of each pattern of multimorbidity in diabetes patients only. Exponentiated coefficients, interpreted as the incident rate ratio for patterns of multimorbidity in diabetes patients compared to the diabetes-only group, were estimated for each model.

Sensitivity analyses were conducted to assess the robustness of the result. First, we adjusted for covariates other than CRP. Second, the raw cognitive function score was used for analysis.

## Results

### Baseline characteristics and sample size

The mean age of the 1,985 participants was 60.6 ± 9.5 years; 53.1% of participants were women. Within the sample, 96 participants (4.8%) were classified as having diabetes only and 1,889 (95.2%) were classified as having multimorbidity. The distribution of baseline covariates is shown in Table 1. The most notable difference in demographics between the two groups was the higher proportion of men in the diabetes-only group. The mean age and BMI were higher in the multimorbidity group, while the educational level was lower. Nearly 81.6% of adults with diabetes have at least two other chronic conditions, and as many as 56.1% have three or more concurrent chronic conditions. The most common chronic diseases in patients with diabetes were dyslipidemia (59.4%), hypertension (54.8%), and depressive symptoms (49.8%).

**Table 1:** Characteristics of the study participants at baseline (wave 1).

Variables	Total (*N* = 1985)	Diabetes only (*N* = 96)	Multimorbidity (*N* = 1889)	*p-*value
*Age (year)*	60.6 (9.5)	57.2 (9.3)	60.8 (9.5)	<0.001*
*Gender*				0.003*
Man	929 (46.8)	59 (61.5)	870 (46.1)	
Woman	1,054 (53.1)	37 (38.5)	1,017 (53.8)	
*Married status*				0.871
Married	1756 (88.5)	86 (89.6)	1,670 (88.4)	
Separated or divorced	15 (0.8)	0	15 (0.8)	
Widowed	202 (10.2)	9 (9.4)	193 (10.2)	
Never married	12 (0.6)	1 (1.0)	11 (0.6)	
*Educational level*				0.029*
Primary school or below	1,309 (65.9)	53 (55.2)	1,256 (66.5)	
Middle school	413 (20.8)	23 (24.0)	390 (20.6)	
High school or above	260 (13.1)	18 (18.8)	242 (12.8)	
*Ever smoking*				0.130
Yes	764 (38.5)	44 (45.8)	720 (38.1)	
No	1,221 (61.5)	52 (54.2)	1,169 (61.9)	
*Ever drinking*				0.003*
Yes	749 (37.7)	50 (52.1)	699 (37.0)	
No	1,230 (62.0)	46 (47.9)	1,184 (62.7)	
*BMI (kg/m^2^)*	24.8 (4.1)	23.5 (3.8)	24.8 (4.1)	0.004*
*HbA1c (%)*	5.50 [5.10, 6.60]	5.50 [5.05, 6.00]	5.60 [5.10, 6.60]	0.044*
*GLU (mg/dL)*	139.7 [127.8, 170.8]	138.1 [129.5, 158.9]	139.5 [127.3, 171.2]	0.902
*BUN (mg/dL)*	15.3 [12.7, 18.3]	14.9 [11.8, 18.1]	15.4 [12.8, 18.5]	0.363
*Cre (mg/dL)*	0.77 [0.67, 0.90]	0.78 [0.66, 0.88]	0.77 [0.66, 0.92]	0.873
*TC (mg/dL)*	197.2 [172.0, 224.6]	190.2 [168.2, 209.7]	197.7 [171.3, 225,5]	0.002*
*TG (mg/dL)*	138.1 [94.7, 218.6]	97.4 [73.0, 128.3]	141.6 [96.5, 222.1]	<0.001*
*HDL (mg/dL)*	44.5 [36.3, 54.5]	52.6[44.8, 63.4]	44.1 [35.6, 53.7]	<0.001*
*LDL (mg/dL)*	115.2 [90.9, 140.7]	112.9 [93.0, 132.2]	114.6 [89.6, 140.8]	0.192
*CRP (mg/dL)*	1.40 [0.70, 3.04]	0.93 [0.51, 2.37]	1.43 [0.70, 3.06]	0.004*
*UA (mg/dL)*	4.43 [3.62, 3.32]	4.47 [3.42, 5.09]	0.44 [3.65, 5.35]	0.148
*Cystatin C (mg/L)*	0.97 [0.83, 1.13]	0.93 [0.85, 1.03]	0.97 [0.83, 1.15]	0.130
*Multimorbidity, N (%)*				
1	366 (18.4)	0 (0.0%)	366 (19.4)	
2	505 (25.4)	0 (0.0%)	505 (26.7)	
3	441 (22.2)	0 (0.0%)	441 (23.3)	
4	307(15.5)	0 (0.0%)	307 (16.3)	
≥5	270 (13.6)	0 (0.0%)	270 (14.3)	
*Chronic diseases, N (%)*				
Dyslipidemia	1,179 (59.4)	0 (0.0%)	1,179 (62.4)	
Hypertension	1,087 (54.8)	0 (0.0%)	1,087 (57.5)	
Depressive symptoms	989 (49.8)	0 (0.0%)	989 (52.4)	
Arthritis	701 (35.3)	0 (0.0%)	701 (37.1)	
Stomach disease	410 (20.7)	0 (0.0%)	410 (21.7)	

BMI, body mass index; HbA1c, glycated hemoglobin; GLU, glucose; Cre, creatinine; BUN, blood urea nitrogen; TC, total cholesterol; TG, triglyceride; HDL, high-density lipoprotein; LDL, low-density lipoprotein; CRP, C-reactive protein; UA, uric acid.

* Significant difference.

### Multimorbidity patterns

**Figure 2:**
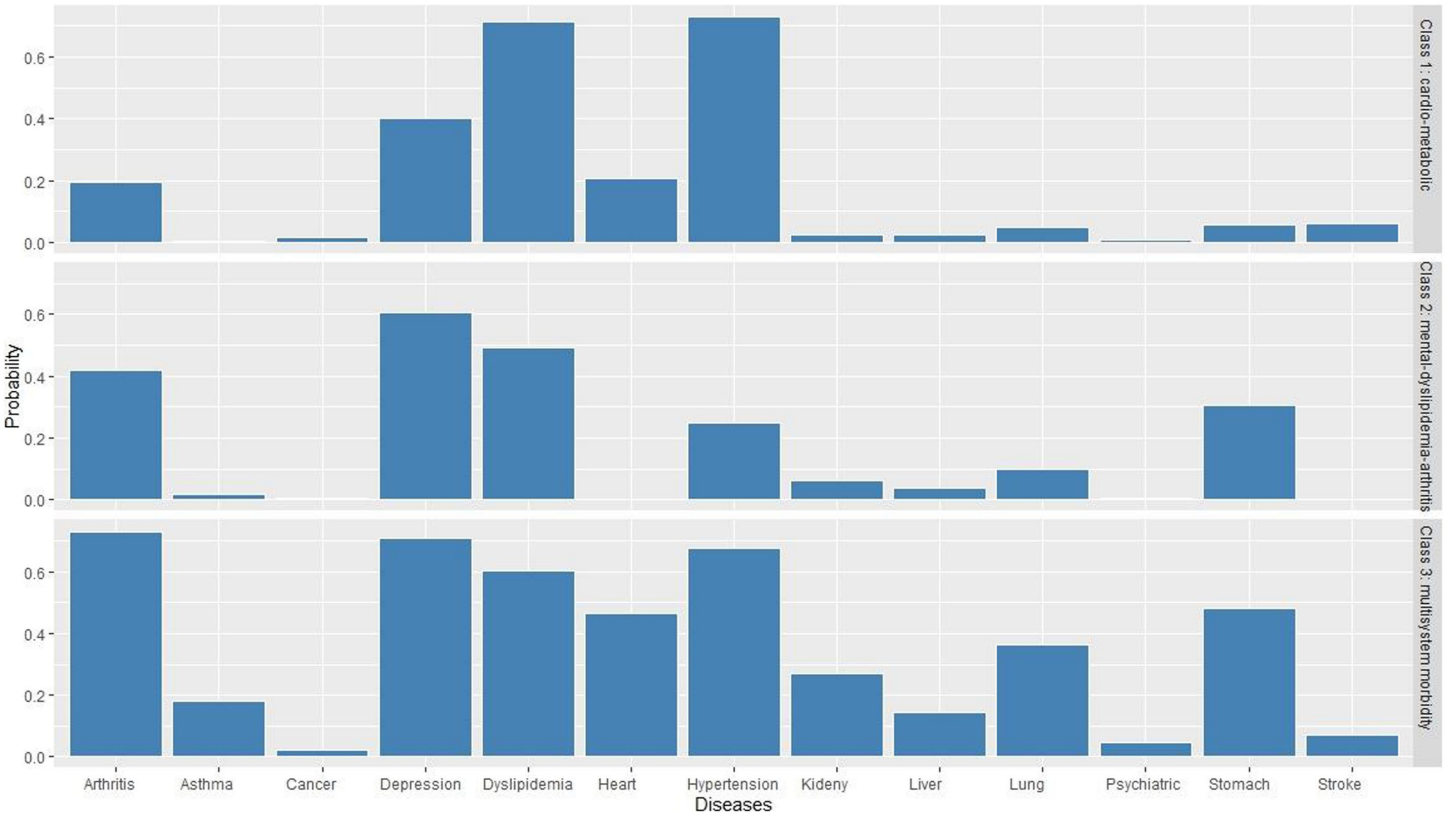
Three-class model of multimorbidity patterns in diabetes patients.

Table 2 displays the BIC, AIC, LMR, entropy values, and proportions of each latent class for a two-class to five-class model. The model finally selected was the three-class model. We presented clusters ordered by descending prevalence and named each latent class according to the most prevalent diseases within each latent class. As shown in Figure 2, class 1 presented excess prevalence of hypertension and dyslipidemia, comprising 51.5% of the total sample, which we named the “cardio-metabolic” class. Class 2 comprised 30.9% of each sample and showed high prevalence of depressive symptoms, dyslipidemia, and arthritis, which we named the “mental-dyslipidemia-arthritis” class. Finally, class 3 presented a higher prevalence of hypertension, dyslipidemia, arthritis, depressive symptoms, and function damage of various organs (chronic lung diseases, heart disease, and stomach or other digestive disease), comprising 17.6% of the sample, which we named the “multisystem morbidity” class.

**Table 2:** Latent class model fit comparison (*n* = 1889).

No of latent classes	Information criteria indices		Classification quality	LMR	Latent classes, *n* (%)
	BIC	AIC	Entropy		
2	19116.5	18966.8	0.59	0.0000	1,517 (80.3)
					372 (19.7)
**3**	**19077.7**	**18850.4**	**0.492**	**0.0022**	**584 (30.9)**
					**972 (51.5)**
					**333 (17.6)**
4	19111.0	18806.0	0.555	0.3186	127 (6.7)
					205 (10.9)
					995 (52.7)
					562 (29.8)
5	19156.7	18774.2	0.559	0.1632	750 (39.7)
					177 (9.4)
					616 (32.6)
					239 (12.7)
					107 (5.7)

Boldface indicates the final selected model.

In the “cardio-metabolic” class, the incidence of hypertension was as high as 73.1%, and the incidence of dyslipidemia was as high as 71.2%. In the “mental-dyslipidemia- arthritis” class, depression (60.4%), dyslipidemia (49.3%), and arthritis (42.0%) had the highest incidence. In the “multisystem morbidity” class, arthritis (72.7%), depression (70.9%), hypertension (67.5), dyslipidemia (60.1%), digestive diseases (48.1%), heart disease (46.5%), and chronic lung diseases (36.4%) had the highest incidence.

### Association between multimorbidity patterns and cognitive function

**Figure 3:**
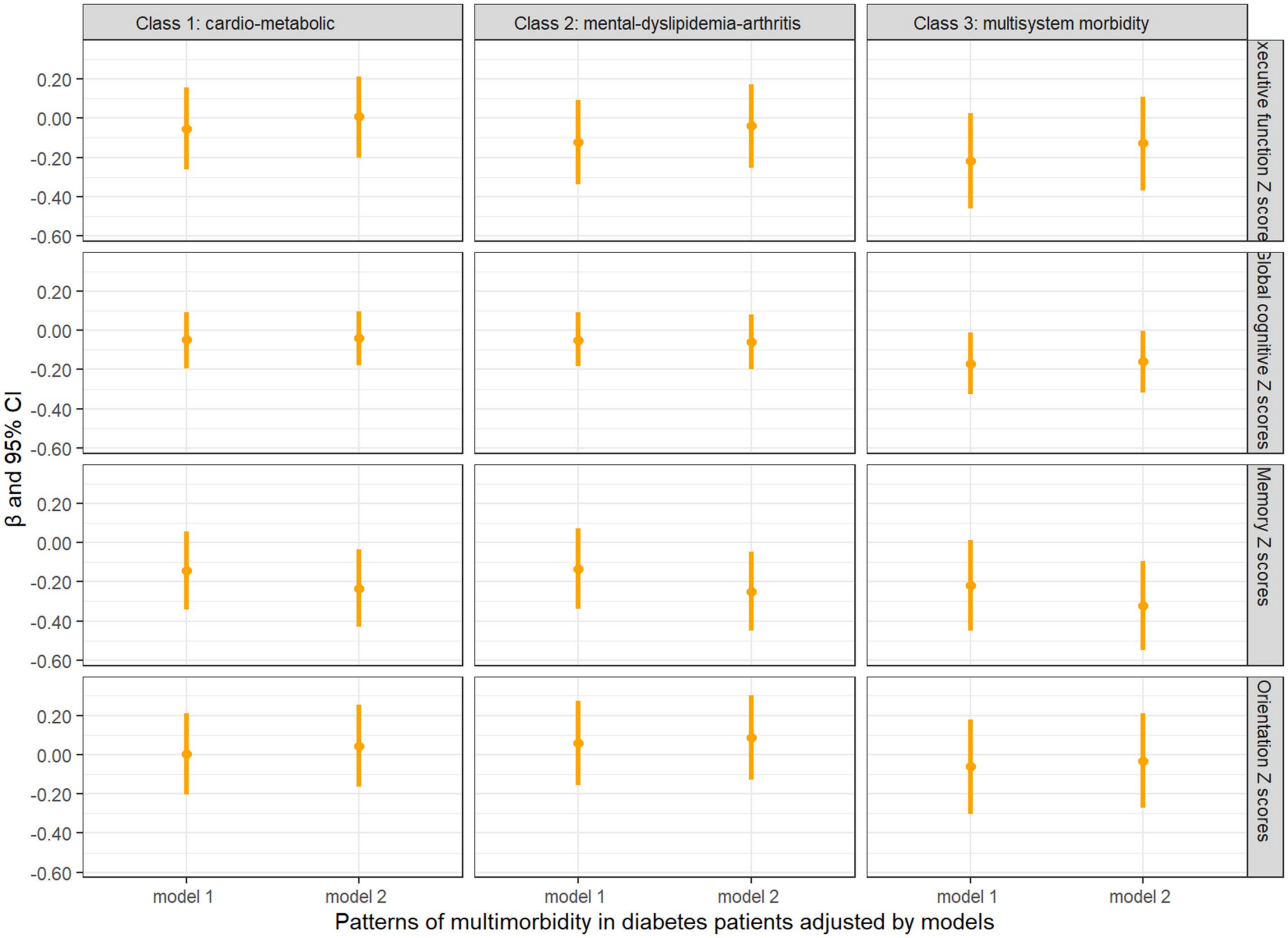
Longitudinal analysis of cognitive z scores comparing baseline diabetes only with multimorbidity patterns in diabetes patients. Model 1: unadjusted covariates. Model 2: adjusted covariates for age, gender, education, marital status, time, smoking, drinking, BMI, BUN, Glu, Cre, HbA1c, TC, HDL, LDL, TG, CRP, UA, and cystatin C.

Figure 3 shows the longitudinal associations between different patterns of multimorbidity in diabetes patients and decline in cognitive Z scores. In unadjusted model 1, none of the cognitive domain Z scores were significantly associated with baseline patterns of multimorbidity in diabetes patients, while the “multisystem morbidity” class had a marginally significant association with decline in global cognitive Z scores (*β* = −0.169, 95% CI = −0.326, −0.012, *p* = 0.0483). In fully adjusted model 2, the “cardio-metabolic” (*β* = −0.233, 95% CI = −0.431, −0.035, *p* = 0.0220), “mental-dyslipidemia-arthritis” (*β* = −0.249, 95% CI = −0.451, −0.047, *p* = 0.0162), and “multisystem morbidity” (*β* = −0.322, 95% CI = −0.550, −0.095, *p* = 0.0058) patterns were significantly associated with decline in memory Z scores. In addition, there was a slight increase in association between “multisystem morbidity” class and decline in global cognitive Z scores (*β* = −0.160, 95%CI = −0.316, −0.004, *p* = 0.0464). We found consistent results in sensitivity analyses (as shown in Supplementary material).

### Association between multimorbidity patterns and physical function

**Figure 4:**
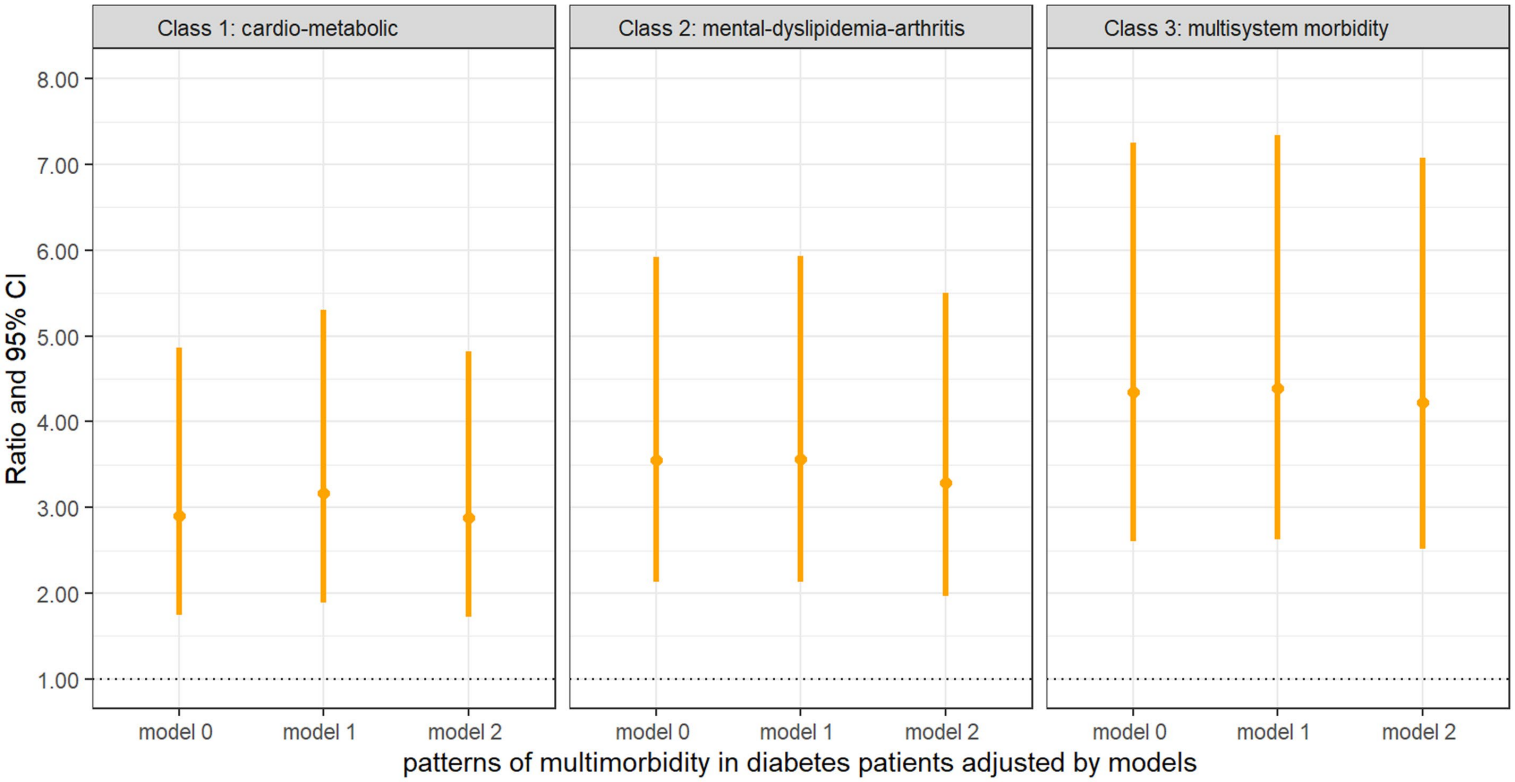
Negative binomial regression of the ADL–IADL index on multimorbidity patterns with diabetes compared with diabetes only Model 0: unadjusted covariates. Model 1: adjusted covariates for age, gender, education, and BMI. Model 2: Model 1 + marital status, smoking, drinking, BUN, Glu, Cre, HbA1c, TC, HDL, LDL, TG, CRP, UA, and cystatin C.

Figure 4 presents comparisons between the ADL–IADL indices of different multimorbidity patterns in diabetes patients and those with diabetes only. All the patterns of multimorbidity in diabetes patients had an association with disability in the unadjusted model and the partially adjusted model that controlled for age, sex, education, and BMI. In fully adjusted models, it was indicated that the “cardio-metabolic” (ratio = 2.88, 95% CI = 1.72, 4.82), “mental-dyslipidemia-arthritis” (ratio = 3.29, 95% CI = 1.97, 5.50), and “multisystem morbidity” (ratio = 4.22, 95% CI = 2.52, 7.08) patterns were still associated with significantly higher ADL–IADL disability compared with patients with diabetes only, and the “multisystem morbidity” class had the highest prospective ADL–IADL index.

## Discussion

Using a large national data set from China, we found the prevalence of multimorbidity in diabetes patients to be 95% and more than half of the adults with diabetes having at least three concurrent chronic conditions. The most observed comorbidities were hypertension, dyslipidemia, and depressive symptoms. We identified three clusters using LCA based on the presence of 13 chronic diseases: the “cardio-metabolic,” “mental-dyslipidemia-arthritis,” and the “multisystem morbidity.” These findings suggested an association between clusters of multimorbidity that span several cardiovascular, physical, psychological, and metabolic systems and memory decline and disability in middle-aged and older adults with diabetes. The difference in populations, definitions, and patterns of multimorbidity makes it difficult to compare the result of the present study. Using a similar definition of multimorbidity, a previous study in the United States found the prevalence of multimorbidity in diabetes patients to be 92% (Kerr et al., 2007), which is similar to the present findings. We also found the prevalence of multimorbidity in diabetes patients to be slightly higher in women than in men, which is consistent with a previous finding (Bing et al., 2023). A potential explanation for the higher prevalence in women is the structural placement of women in society (Arber and Cooper, 1999).

A systematic review of 12 studies on multimorbidity among patients with diabetes found similarities for three types of condition clusters, namely, cardiometabolic precursor conditions, vascular conditions, and mental health conditions (Cicek et al., 2021). In contrast, the present study found three main clusters: the first containing hypertension and dyslipidemia, with a higher prevalence; the second dominated by depressive symptoms, dyslipidemia and arthritis; the third consisting of functional impairment of organs, in addition to hypertension, dyslipidemia, depressive symptoms, and arthritis, with a lower prevalence. The cluster of mental health, arthritis, and dyslipidemia had not been observed in previous studies. There are several possible explanations. First, individuals with diabetes reported higher levels of depressive symptoms (Anderson et al., 2001). The risks of comorbid arthritis were significantly higher in the presence of concomitant depressive symptoms, among both diabetic and non-diabetic individuals (Black, 1999). Second, arthritis leads to limited joint movement, impaired mobility (Rodriguez et al., 2022), loss of control over the original life, and being prone to depressive symptoms (Mammen and Faulkner, 2013; Schuch et al., 2018). Third, arthritis is a chronic inflammation, and some inflammatory factors may be involved in the body’s normal lipid metabolism. The tumor necrosis factor (TNF) increases the fat decomposition, resulting in the increase of the level of the cyclic free fatty acid, which stimulates the production of triglycerides of the liver, which causes TNF-induced hyperlipemia (Feingold et al., 1992).

When we examined the association of multimorbidity patterns in diabetes patients with cognitive function, we found a “multisystem morbidity” class that consists of several chronic conditions, particularly depressive symptoms, is associated with declines in global cognitive function, which aligns with previous results (de Araujo et al., 2022). In addition, we found that all patterns of multimorbidity in diabetes patients were associated with memory decline. Similar to this result, a longitudinal cohort study in the United Kingdom showed that individuals with certain combinations of health conditions are more likely to have lower levels of memory compared to those with no multimorbidity, and their memory scores tend to differ between combinations (Bendayan et al., 2021). Several mechanisms may explain the link between multimorbidity in diabetes patients and impaired memory. First, multimorbidity can affect the ability of diabetes patients to engage in self-management activities, resulting in suboptimal diabetes control, which in turn can impact memory function (Ryan et al., 2006; Kielstein, 2013). Second, diabetes patients often experience vascular changes, such as arteriosclerosis, that can affect blood flow to the brain. These changes in blood supply can lead to alterations in brain structure and function, ultimately affecting memory (Cukierman et al., 2005; Biessels and Reijmer, 2014). Third, inflammatory mediators associated with multimorbidity can progressively affect both microvascular and macrovascular structures, leading to structural changes that impair the ability to retain long-term memory. Finally, the presence of multiple comorbidities can have a negative impact on the patient’s mental well-being, including increased levels of anxiety and depression, which can interfere with cognitive processes, including memory function.

With regard to physical function, we found that multimorbidity was significantly associated with an increased risk of disability, which was consistent with previous research studies (Quiñones et al., 2016, 2018; Wei et al., 2018; Pengpid et al., 2022). We also found that the “cardio-metabolic” and “multisystem morbidity” classes were associated with a higher risk of disability, which is consistent with previous studies showing a positive association of metabolic multimorbidity with a higher risk of disability (Zhao et al., 2021). Additionally, the present study included patients with diabetes, whereas most included all patients with multimorbidity. A similar cohort study was conducted among US participants, though the magnitude of this association differed from this study (i.e., exponential coefficients ranging from 3.99 to 18.15, in comparison to our 2.88 to 4.22) (Quiñones et al., 2019). However, our findings do correspond with evidence found in Mexican older adults (McClellan et al., 2021). We speculate that the difference might be interpreted by the following reasons. First, multimorbidity with diabetes is classified in different ways, one is a specific comorbidity, and the other is an overall disease group. Second, there are differences in demographic characteristics, socioeconomic status, and lifestyle factors. Previous studies have shown that age, sex, material status, income, education, smoking, and alcohol consumption are associated with morbidity and disability (Jindai et al., 2016; Berlinski et al., 2021).

### Strengths and limitations

To our knowledge, this is the first study using data from a nationally representative sample among Chinese middle-aged and older adults to identify patterns of multimorbidity in diabetes by a list of 13 chronic conditions and to explore the effect on physical and cognitive function. However, some limitations need to be acknowledged. First, our study could not capture all of chronic conditions, and different chronic diseases may generate different numbers of clusters and constituents within the clusters. Future studies should use datasets that cover various chronic conditions to better explain the multimorbidity patterns in diabetes patients. Second, these chronic diseases were diagnosed based on self-reported information, which may be under- or over-reported, and can thus be subject to measurement errors or lack of accuracy. Third, although we have adjusted some potential confounders based on previous research studies, some extra confounders were not considered, such as physical activity. Fourth, our study did not take into account the effect of self-management level, which results in suboptimal diabetes control on cognitive function and physical function in diabetes patients. Finally, the assessment of cognitive function used in the present study may not be the most commonly used approach, although the items in CHARLS similar to those used in the US Health and Retirement Study.

## Conclusion

Our findings indicated that most patients with diabetes have a multimorbidity, and multimorbidity patterns were associated with increased risk of memory decline and disability. These findings suggest health practitioners should pay special attention to early detection of physical and mental health among middle-aged and older adults with diabetes. Moreover, advocating for public awareness of the potentially increased risk of memory decline and disability among diabetes patients with multimorbidity is necessary as a preventive approach for their physical and mental health.

## Data availability statement

Publicly available datasets were analyzed in this study. This data can be found at: http://charls.pku.edu.cn/en.

## Ethics statement

The studies involving humans were approved by the Biomedical Ethics Committee of Peking University. The studies were conducted in accordance with the local legislation and institutional requirements. The human samples used in this study were acquired from China Health and Retirement Longitudinal Study (CHARLS) is an ongoing nationally representative survey to investigate the social, economic and health status of middle-aged and elderly people aged 45 years and above in China. All participants were required to sign informed consent.

## Author contributions

XZ: Data curation, Formal analysis, Software, Writing – original draft, Writing – review & editing. JQ: Conceptualization, Data curation, Methodology, Writing – review & editing, Writing – original draft. HL: Data curation, Investigation, Writing – original draft. JC: Investigation, Writing – original draft. QZ: Supervision, Writing – review & editing. XY: Supervision, Writing – review & editing.
