# Supplementary material for: The effect of multimorbidity patterns on physical and cognitive function in diabetes patients: a longitudinal cohort of middle-aged and older adults in China
Source: Front Aging Neurosci. 2024 May 14;16:1388656. doi: 10.3389/fnagi.2024.1388656 (PMC11130586; doi:10.3389/fnagi.2024.1388656)
Supplement: Supplementary file 1 [file Table_1.DOC]

**Supplementary Table1：Longitudinal analysis of** **cognitive z scores comparing baseline diabetes only and multimorbidity patterns in diabetes patients, using linear mixed models**

|  | **Diabetes-only** | **Patterns of multimorbidity** | | |
| --- | --- | --- | --- | --- |
|  |  | **class1：metabolic** | **class2：mental-**  **dyslipidemia-arthritis** | **class3：arthritis- mental- metabolic** |
| **Global cognitive z score** | |  |  |  |
| Model 1 | 0.000(ref.) | -0.05(-0.194, 0.091) | -0.05(-0.184, 0.090) | -0.17(-0.326, -0.012) * |
| Model 2 | 0.000(ref.) | -0.04(-0.177, 0.094) | -0.06(-0.201, 0.077) | -0.16(-0.315, -0.003) * |
| **Memory z score** | |  |  |  |
| Model 1 | 0.000(ref.) | -0.14(-0.342, 0.056) | -0.13(-0.339, 0.071) | -0.22(-0.450, 0.011) |
| Model 2 | 0.000(ref.) | -0.23(-0.430, -0.034) * | -0.25(-0.453, -0.049) * | -0.32(-0.548, -0.093) ** |
| **Executive function z score** | |  |  |  |
| Model 1 | 0.000(ref.) | -0.05(-0.263, 0.155) | -0.12(-0.338, 0.094) | -0.22(-0.461, 0.024) |
| Model 2 | 0.000(ref.) | 0.006(-0.201, 0.213) | -0.04(-0.250, 0.174) | -0.129(-0.369, 0.109) |
| **Orientation z score** | |  |  |  |
| Model 1 | 0.000(ref.) | 0.01(-0.202, 0.212) | 0.06(-0.155, 0.273) | -0.06(-0.301, 0.177) |
| Model 2 | 0.000(ref.) | 0.05(-0.164, 0.255) | 0.09(-0.129, 0.301) | -0.03(-0.270, 0.212) |

Model 1：Unadjusted covariates.

Model 2：adjusted covariates for age, gender, education, marital status, time, smoking, drinking, BMI, BUN, Glu, Cre, HbA1c, TC, HDL, LDL, TG, UA, Cystatin C.

*: P＜0.05；**: P＜0.01.

**Supplementary Table2：Longitudinal analysis of row cognitive function scores comparing baseline diabetes only and multimorbidity patterns in diabetes patients, using linear mixed models**

|  | **Diabetes-only** | **Patterns of multimorbidity** | | |
| --- | --- | --- | --- | --- |
|  |  | **class1：metabolic** | **class2：mental-**  **dyslipidemia-arthritis** | **class3：arthritis- mental- metabolic** |
| **Global cognitive z score** | |  |  |  |
| Model 1 | 0.000(ref.) | -0.36(-1.148, 0.435) | -0.39(-1.214, 0.425) | -0.91(-1.832, 0.006) |
| Model 2 | 0.000(ref.) | -0.52(-1.304, 0.258) | -0.70(-1.504, 0.096) | -1.14(-2.046, -0.244) * |
| **Memory z score** | |  |  |  |
| Model 1 | 0.000(ref.) | -0.44(-1.071, 0.174) | -0.42(-1.062, 0.223) | -0.69(-1.408, 0.034) |
| Model 2 | 0.000(ref.) | -0.73(-1.348, -0.108) * | -0.78( -1.413, -0.148) * | -1.01(-1.721, -0.296) ** |
| **Executive function z score** | |  |  |  |
| Model 1 | 0.000(ref.) | -0.08(-0.384, 0.226) | -0.18(-0.494, 0.137) | -0.32(-0.672, 0.035) |
| Model 2 | 0.000(ref.) | 0.09(-0.294, 0.311) | -0.06(-0.368, 0.252) | -0.19(-0.537, 0.159) |
| **Orientation z score** | |  |  |  |
| Model 1 | 0.000(ref.) | 0.004(-0.151, 0.158) | 0.04(-0.116, 0.205) | -0.05(-0.226, 0.133) |
| Model 2 | 0.000(ref.) | 0.03(-0.125, 0.190) | 0.07(-0.095, 0.227) | -0.02(-0.204, 0.158) |

Model 1：Unadjusted covariates.

Model 2：adjusted covariates for age, gender, education, marital status, time, smoking, drinking, BMI, BUN, Glu, Cre, HbA1c, TC, HDL, LDL, TG, CRP, UA, Cystatin C.

*: P＜0.05；**: P＜0.01.
